# DNA damage response at telomeres contributes to lung aging and chronic obstructive pulmonary disease

**DOI:** 10.1152/ajplung.00293.2015

**Published:** 2015-09-18

**Authors:** Jodie Birch, Rhys K. Anderson, Clara Correia-Melo, Diana Jurk, Graeme Hewitt, Francisco Madeira Marques, Nicola J. Green, Elizabeth Moisey, Mark A. Birrell, Maria G. Belvisi, Fiona Black, John J. Taylor, Andrew J. Fisher, Anthony De Soyza, João F. Passos

**Affiliations:** ^1^Newcastle University Institute for Ageing, Institute for Cell and Molecular Biosciences, Newcastle upon Tyne, United Kingdom;; ^2^Lung Immunobiology Group, Institute of Cellular Medicine, Newcastle University, Newcastle upon Tyne, United Kingdom;; ^3^Institute of Transplantation, Freeman Hospital, Newcastle upon Tyne, United Kingdom;; ^4^Institute of Cellular Medicine, Newcastle University, Newcastle upon Tyne, United Kingdom;; ^5^Respiratory Pharmacology, Airway Disease Section, National Heart and Lung Institute, Faculty of Medicine, Imperial College London, London, United Kingdom; and; ^6^Department of Pathology, Newcastle upon Tyne Hospitals Trust, Newcastle upon Tyne, United Kingdom

**Keywords:** senescence, airway epithelial cells, cigarette smoke

## Abstract

Cellular senescence has been associated with the structural and functional decline observed during physiological lung aging and in chronic obstructive pulmonary disease (COPD). Airway epithelial cells are the first line of defense in the lungs and are important to COPD pathogenesis. However, the mechanisms underlying airway epithelial cell senescence, and particularly the role of telomere dysfunction in this process, are poorly understood. We aimed to investigate telomere dysfunction in airway epithelial cells from patients with COPD, in the aging murine lung and following cigarette smoke exposure. We evaluated colocalization of γ-histone protein 2A.X and telomeres and telomere length in small airway epithelial cells from patients with COPD, during murine lung aging, and following cigarette smoke exposure in vivo and in vitro. We found that telomere-associated DNA damage foci increase in small airway epithelial cells from patients with COPD, without significant telomere shortening detected. With age, telomere-associated foci increase in small airway epithelial cells of the murine lung, which is accelerated by cigarette smoke exposure. Moreover, telomere-associated foci predict age-dependent emphysema, and late-generation *Terc* null mice, which harbor dysfunctional telomeres, show early-onset emphysema. We found that cigarette smoke accelerates telomere dysfunction via reactive oxygen species in vitro and may be associated with ataxia telangiectasia mutated-dependent secretion of inflammatory cytokines interleukin-6 and -8. We propose that telomeres are highly sensitive to cigarette smoke-induced damage, and telomere dysfunction may underlie decline of lung function observed during aging and in COPD.

chronic obstructive pulmonary disease (COPD) is a major global health problem that is becoming increasingly prevalent (26). COPD is characterized by chronic inflammation of the peripheral airways and lung parenchyma and involves airway fibrosis, mucous hypersecretion (chronic bronchitis), and destruction of alveolar air spaces (emphysema). The key risk factor for COPD is cigarette smoking (19).

Accelerated lung aging and cellular senescence have been associated with COPD (38, 48). Senescence, defined as the irreversible loss of division potential in somatic cells, plays important roles in vivo: on the one hand, it protects against cancer progression, yet on the other hand it contributes to age-dependent tissue dysfunction (10). Evidence is mounting that cells bearing senescent markers accumulate in tissues with age (23) and in age-related diseases (13).

Telomeres are specialized structures at the ends of chromosomes consisting of tandem TTAGGG repeats stabilized by a complex of proteins, known as shelterin (15). Shelterin is thought to arrange telomeric DNA into a loop structure known as the T-loop. It is believed that, during replicative senescence, the progressive loss of telomere repeats destabilizes T-loops, increasing the probability of telomere uncapping, i.e., loss of shelterin (21). Telomere uncapping, whether by inhibition of shelterin or telomere shortening due to extensive replication, has been shown to activate the DNA damage response (DDR) in a manner similar to double-strand breaks (DSBs) (14). Uncapped telomeres become associated with DDR factors, such as phosphorylated forms of the histone protein 2A.X (H2A.X) and ataxia telangiectasia mutated (ATM), which can activate a signaling cascade leading to culmination of senescence (14). More recently, it has been shown that a DDR can induce senescence, irrespective of telomere length, which has been attributed to telomeres being particularly susceptible to oxidation-induced damage and to the inability of telomeres to repair DSBs (20, 25, 30). Moreover, it has been shown in vivo that, with age, telomeres colocalizing with DDR proteins increase in the skin of baboons (23) and in the liver, brain, and gut of mice, which can occur irrespectively of length (20, 25).

Telomere shortening has been associated with COPD in circulating leukocytes (45), alveolar epithelial cells (27, 36), and pulmonary vascular endothelial cells (5). However, it is unclear whether activation of a DDR at telomeres contributes to senescence and tissue dysfunction in the aging lung and to COPD-associated accelerated lung aging. In our study, we investigate the role of telomere dysfunction in the aging mouse lung and its potential role in cigarette smoke-induced COPD.

## METHODS

### 

#### Study subjects.

Patients undergoing lung resection for localized lung tumors were recruited as controls from the Freeman Hospital, Newcastle upon Tyne, UK (Table 1). Samples from patients with advanced COPD were obtained from an archive of explant lung tissue taken at the time of lung transplantation at the Freeman Hospital. A smaller number of cases were used for immunofluorescence in situ hybridization (immuno-FISH) analysis due to limited availability of tissue at time of staining. All samples were parenchymal, and only airways with a diameter of <2 mm and without cartilage were included in the analysis. The clinical characteristics of these subjects are the same as those listed in Table 1. All subjects gave written, informed consent before inclusion in the study. This work was approved by the County Durham and Tees Valley 2 Research Ethics Committee (Res-11/NE/0291).

**Table 1.** Clinical characteristics of patients with COPD and controls (tissue samples)

	Patients with COPD	Controls
*n*	19	11
Sex, male/female	11/8	3/8
Age, yr	52.84 ± 6.9†	70.45 ± 7.56
FEV_1_, liter	0.53 ± 0.22†	1.79 ± 0.32
FEV_1_, %	17.63 ± 6.99†	84.36 ± 9.45
FVC, liter	1.89 ± 0.51*	2.69 ± 0.59
FVC, %	52.05 ± 14.15†	95.9 ± 16.8
Smoking history, pack·yr	39.95 ± 27.1	31.3 ± 17.93
GOLD score, I/II/III/IV	0/0/1/18	0/0/0/0

Values are means ± SD; *n*, no. of subjects. COPD, chronic obstructive pulmonary disease; FEV_1_, forced expiratory volume in 1 s; FEV_1_ %, percentage of predicted FEV_1_; FVC, forced vital capacity; FVC %, percentage of predicted FVC; GOLD, Global Initiative for Chronic Obstructive Lung Disease.

* *P* < 0.001,

† *P* < 0.0001 compared with controls.

#### Animals.

Wild-type C57BL/6 male mice were used [*n* = 3–5 per age group (6.5, 15 and 24 mo)]. *TERC*^−/−^ C57BL/6 male mice were bred to produce successive generations of mice with decreasing telomere length. Lungs from fourth-generation (G4) mice were collected. All work was compiled with the guiding principles for the care and use of laboratory animals. The project was approved by the Faculty of Medical Sciences Ethical Review Committee, Newcastle University (project license no. 60/3864).

Lung tissues from mice exposed to either room air or cigarette smoke were a kind gift from Dr. Mark Birrell, Imperial College London, UK. Male C57BL/6 mice (*n* = 5/group) at 10 wk of age were subjected to a whole body cigarette smoke exposure system or room air, as previously described (18). Briefly, cigarette smoke was generated using 3R4F cigarettes (cigarette filter removed, Tobacco Health Research Institute, University of Kentucky, Lexington, KY) and pumped into a Teague chamber (136 liters) for 1 h, twice daily (500 ml/min), for 14 days. Mice were killed 24 h after the final exposure.

#### Cell culture and treatments.

Human embryonic lung MRC5 fibroblasts were obtained from European Collection of Cell Cultures (Salisbury, UK) and cultured in Dulbecco's modified Eagle's medium (DMEM) (Sigma, Dorset, UK), supplemented with fetal bovine serum (10% vol/vol), l-glutamine (2 mM), and penicillin/streptomycin and maintained at 37°C, 5% CO_2_.

Primary human small airway epithelial cells were isolated from bronchial brushings carried out during research bronchoscopy (normal controls) or from explant lung tissue specimens (COPD) (Table 2). The work was performed under approval of the Newcastle 1 Research Ethics Committee. Primary human bronchial epithelial cells were cultured on 0.5% Purecol-coated (Invitrogen, Carlsbad, CA) dishes in small airway epithelial cell growth medium (L/SABM), supplemented with 2% fetal bovine serum, 100 U/ml penicillin, and 100 mg/ml streptomycin (Lonza, Basel, Switzerland).

**Table 2.** Clinical characteristics of patients with COPD and controls (small airway epithelial cells)

	Patients with COPD	Normal Controls
*n*	5	5
Sex, male/female	4/1	4/1
Age, yr	56 ± 6.9	50.4 ± 6.9
FEV_1_, liter	0.53 ± 0.2*	3.59 ± 1.1
FEV_1_, %	16 ± 4.5†	106.8 ± 17.7
FVC, liter	2.57 ± 1.1	4.9 ± 1.7
FVC, %	61.6 ± 20.8*	118.2 ± 23.3
Smoking history, pack·yr	40.4 ± 23.7*	0
GOLD score, I/II/III/IV	0/0/0/0/5	0/0/0/0

Values are means ± SD; *n*, no. of subjects.

* *P* < 0.05,

† *P* < 0.01 compared with controls.

MRC5 fibroblasts (population doublings 20–25) were grown until replicative senescence and cultured with DMEM plus 5% cigarette smoke extract (CSE) or DMEM alone. CSE was generated by bubbling smoke from one research-grade cigarette (University of Kentucky; 4A1) into 25 ml DMEM. The solution was filtered (0.2 μm), and the resulting CSE designated 100%. The CSE solution was diluted to 5% in sterile DMEM and used immediately. CSE or DMEM alone was replenished every 48 h. Identical experiments under hypoxic conditions (3% O_2_) were run in parallel. Human primary small airway epithelial cells (*passages 1–3*) were treated with two exposures of 5% CSE or media alone (control), 48 h apart.

Chemical inhibitors used were KU55933 (ATM chemical inhibitor) (10 μM, diluted in DMSO) (R&D, 3544). Inhibitors were replaced every 48 h, along with 5% CSE or fresh DMEM.

#### Immunofluorescence.

Cells grown on coverslips were fixed with 2% paraformaldehyde, permeabilized with PBG-Triton and incubated with the primary antibody at 4°C overnight. The following day, cells were incubated with fluorescein-conjugated secondary antibody (Alexa Fluor 488 or 594; Invitrogen) for 45 min at room temperature. Primary antibodies used were as follows: rabbit polyclonal anti-Ki67 (ab15580; 4 μg/ml Abcam), mouse monoclonal anti-γH2A.X (no. 05–636; 0.25 μg/ml Millipore), and mouse monoclonal anti-p16 (SC-81156; 1:500 Santa Cruz).

#### Immuno-FISH.

Immuno-FISH was performed as described (25). Briefly, cells grown on sterile coverslips were fixed with 2% paraformaldehyde and incubated with anti-γH2A.X antibody (mouse monoclonal, no. 05–636, Millipore) overnight at 4°C. After application of the secondary antibody, cells were washed with PBS, and FISH was performed. Ten microliters of Cy-3-labeled telomere-specific (C3TA2)3 peptide nucleic acid probe (4 ng/μl) (Panagene) was applied to the cells, followed by denaturation at 80°C and hybridization for 2 h at room temperature in the dark. Cells were washed three times for 10 min with wash buffer [70 ml formamide (70%), 30 ml saline-sodium citrate 2%] and three times for 5 min with Tris-buffered saline-Tween 0.05%. Cells were incubated with 4,6-diamidino-2-phenylindole (DAPI), mounted, and imaged using a Leica DM5500B fluorescence microscope. In depth *Z*-stacking was used (images were captured as stacks separated by 0.247 μm with ×100 objective) followed by Huygens (SVI) deconvolution.

For immuno-FISH in formalin-fixed, paraffin-embedded murine and human lung tissues, sections were dewaxed in 100% Histoclear and hydrated in 100, 90, and 70% ethanol (2 × 5 min incubations) and in distilled water (2 × 5 min). For antigen retrieval, the slides were placed in 0.01 M citrate buffer and heated until boiling for 10 min. After cooling down to room temperature, the slides were washed twice with distilled water for 5 min. After blocking in normal goat serum (1:60) in BSA/PBS, primary antibody (rabbit polyclonal anti-γH2AX 1:400) (Cell Signaling, 9718) was applied and incubated at 4°C overnight. The next day, slides were washed three times in PBS, incubated with secondary antibody for 30 min, washed three times in PBS, and incubated with Avidin DCS (1:500) for 20 min. Following incubation, slides were washed three times in PBS and dehydrated with 70, 90, and 100% ethanol for 3 min each. Sections were denatured for 5 min at 80°C in hybridization buffer [70% formamide (Sigma), 25 mM MgCl_2_, 1 M Tris, pH 7.2, 5% blocking reagent (Roche)] containing 2.5 μg/ml Cy-3-labeled telomere specific (CCCTAA) peptide nuclei acid probe (Panagene), followed by hybridization for 2 h at room temperature in the dark. The slides were washed twice, for 15 min each, with wash buffer, followed by 2× saline-sodium citrate and PBS washes for 10 min. Sections were incubated with DAPI, mounted, and imaged. In depth *Z*-stacking was used (a minimum of 40 optical slices with ×100 objective) followed by Huygens (SVI) deconvolution.

#### Immunohistochemistry.

Sections were dewaxed in xylene (5 min), rehydrated through graded ethanol solutions (100, 90, and 70%) and washed in distilled H_2_O. Endogenous peroxidase activity was blocked by immersing sections in 0.3% H_2_O_2_ (Sigma, H1009) diluted in methanol for 30 min. To retrieve antigens, sections were boiled in 0.01 M citrate (pH 6.0) or 0.01 M EDTA buffer (pH 8.0). Sections were blocked in 5% nonfat milk protein diluted in Tris-buffered saline (human) or normal goat serum diluted 1:60 in 0.1% BSA in PBS (mouse). Sections were incubated with the primary antibody overnight at 4°C or for 45 min to 1 h at room temperature. For human tissue, anti-mouse (Dako, K4006) or anti-rabbit (Dako, K4010) secondary antibody conjugated with a horseradish-peroxidase-labeled polymer was added (Envision+ System-HRP, Dako), and slides were treated with 3,3′-diaminobenzidine for 5–10 min. For mouse tissue, biotinylated secondary antibody was added and detected using the rabbit peroxidase ABC kit (Vector Laboratories, PK-4001), according to the manufacturer's instructions. Substrate was developed using the NovaRed kit (Vector Laboratories, SK-4800). Nuclei were counterstained with Carrazzi hematoxylin, and sections were dehydrated through graded ethanol solutions, cleared in xylene, and mounted in di-*n*-butyle phthalate in xylene (Thermo Scientific, LAMB-DPX). Staining was analyzed with a NIKON ECLIPSE-E800 microscope, and images were captured with a Leica DFC420 camera using the LAS software (Leica). Primary antibodies used were as follows: mouse monoclonal anti-p16 (1:500, SC-81156; Santa Cruz), rabbit polyclonal anti-SIRT1 (1:100, ab13749; Abcam), and rabbit polyclonal anti-p21 (1:200, ab7960; Abcam).

#### Senescence-associated β-galactosidase staining.

Senescence-associated β-galactosidase (Sen-β-Gal) staining was carried out as previously described (17). Briefly, cells were fixed with 2% formaldehyde in PBS for 5 min. Following fixation, cells were washed once with PBS and incubated at 37°C for 16 h in freshly prepared Sen-β-Gal staining solution containing 2 mM magnesium chloride, 150 mM sodium chloride, 40 mM citric acid, 12 mM sodium phosphate dibasic, 5 mM potassium ferrocyanide, 5 mM potassium ferricyanide, and 1 mg/l, 5-bromo-4-chloro-3-inolyl-β-d-galactoside at pH 6.0. Following staining, nuclei were stained with DAPI, and cells were imaged.

#### Analysis of telomere length by real-time PCR.

Telomere length of isolated epithelial cells (Table 3) was measured by quantitative real-time PCR, as described (34). Telomere length was measured as abundance of telomeric template vs. a single gene by quantitative real-time PCR. Measurements were performed in quadruplicate. Three DNA samples with known telomere lengths (3.0, 5.5, and 9.5 kilobase pairs) were run as internal standards, allowing us to estimate telomere length in base pairs.

#### Detection of ROS levels.

Superoxide anion levels were determined by flow cytometric analysis of MitoSOX fluorescence, as described (40).

#### Detection of senescence-associated secretory phenotype factors.

A Quantibody human cytokine array for 20 cytokines (RayBiotech; QAH-CYT-1) was performed. Concentrations of interleukin (IL)-6 and IL-8 in cell culture media were determined using sandwich ELISA (R&D Systems; DY206/DY208), according to the manufacturer's instructions. Limits of detection for these assays were 10 pg/ml.

#### Western blotting.

Western blotting was conducted using routine protocol.

#### Statistical analysis.

Data are represented as means ± SE or median ± range. Where data were normally distributed, statistically significant differences between groups were assessed using ANOVA, and significant differences between two groups were evaluated using an independent samples *t*-test. Where data were not normally distributed, statistically significant differences between groups were assessed using the Kruskal Wallis test, and significant differences between two groups were evaluated using the Mann-Whitney *U*-test. *P* values <0.05 were considered significant. Data were analyzed with GraphPad Prism version 6.0, GraphPad Software, San Diego, CA (www.graphpad.com).

## RESULTS

### 

#### Patients with COPD show increased telomere-associated foci in small airway epithelial cells.

**Fig. 1.**
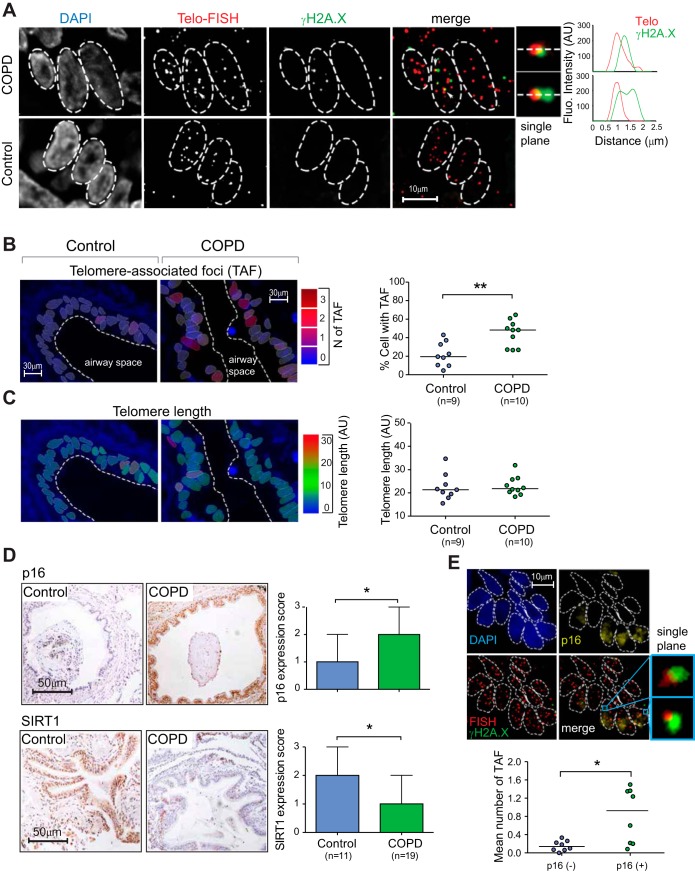
Chronic obstructive pulmonary disease (COPD) patients show increased telomere-associated DNA damage foci (TAF) in small airway epithelial cells without significant telomere shortening. Explant lung tissue sections from patients with COPD and lung resection specimens from control subjects containing small airway material were analyzed for expression of senescence-associated markers by immunofluorescence in situ hybridization (immuno-FISH) and immunohistochemistry. *A*: representative images of immuno-FISH staining for γ-histone protein 2A.X (γH2A.X; green) and telomeres (red) in small airway epithelial cells from patients with COPD and controls captured using ×100 oil objective and following Huygens (SVI) deconvolution. Arrows point to γH2A.X foci colocalizing with telomeres (TAF), depicted by associated histograms and shown at higher magnification on the *right* (images are from one single *Z*-plane). *B*: immuno-FISH images of small airway epithelium in patients with COPD and controls color-coded according to number of TAF (blue: low number; red: high number). *C*: quantitative FISH (Q-FISH) images of small airway epithelium in patients with COPD and controls color-coded according to telomere length (blue: short; red: long). Dot plots represent percentage of cells positive for TAF and mean telomere length for each individual generated by quantifying *Z*-stacks of at least 50 cells per subject. The horizontal line represents group median. *D*: representative images of immunostaining for p16 and SIRT1 (brown) in small airway epithelial cells captured using ×40 objective. Arrows point to positive cells. Bar graphs represent the level of positive staining in the airway epithelium quantified using a semiquantitative scoring method. Values are median + range. *E*: representative image of double immunofluorescence staining for γH2A.X (green) and p16 (yellow) combined with Q-FISH (red), carried out on lung tissue samples from patients with COPD (*n* = 8) to determine whether TAF and p16 expression colocalizes. Dot plot represents mean number of TAF in both p16-positive and p16-negative cells per individual, with the horizontal line representing group median. AU, arbitrary units. Statistics: Mann-Whitney *U*-test: *P < 0.05, **P < 0.01.

**Fig. 2.**
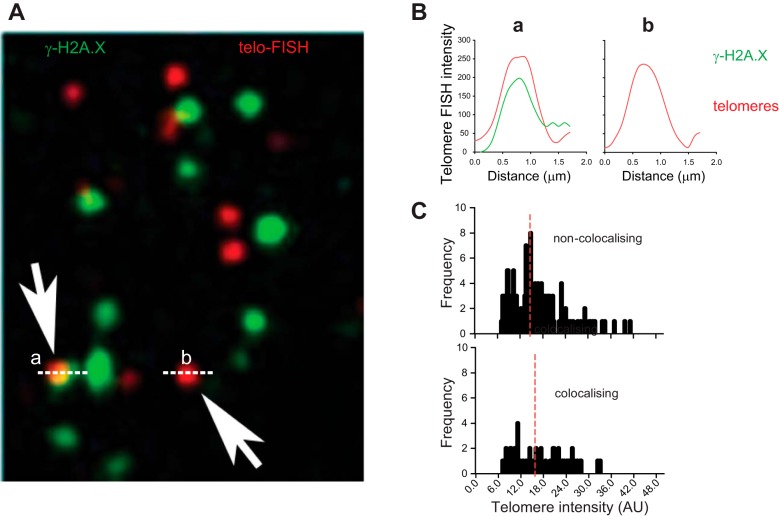
TAF in COPD occur irrespective of telomere length. *A*: representative immuno-FISH (γH2A.X and telomere peptide nucleic acid probe) of small airway epithelial cell present in COPD lung tissue. Arrows indicate telomeres of similar length colocalizing (*a*) or not (*b*) with γH2A.X. *B*: quantification of telomere intensity of colocalizing (*a*) or non-colocalizing (*b*) telomere. *C*: quantification of telomere intensity in colocalizing and non-colocalizing telomeres in COPD patients; red line indicates median telomere intensity. Five hundred individual telomeres were quantified per condition. Mann-Whitney *U*-test shows no statistically significant difference.

To assess telomere dysfunction, we obtained explant lung tissue from patients undergoing transplantation for COPD (*n* = 10) and from controls (*n* = 9) undergoing pulmonary resection for localized lung cancer (Table 1). We performed telomere specific quantitative FISH (Q-FISH), together with immunofluorescence staining against DNA damage protein γH2A.X (immuno-FISH). Analysis revealed a significant increase in percentage of small airway epithelial cells containing telomere-associated DNA damage foci (TAF) in patients with COPD (Fig. 1, *A* and *B*). No significant differences in telomere FISH intensity were detected (Fig. 1*C*). Similarly, analysis of individual telomeres in small airway epithelial cells in the COPD lung revealed no differences in FISH intensity between colocalizing (with γH2A.X) and non-colocalizing telomeres (Fig. 2), suggesting that TAF can occur independently of telomere length. To determine whether other senescence markers were increased in patients with COPD, we conducted immunohistochemistry against p16, p21, and SIRT1. p16 and p21 are cyclin-dependent kinase inhibitors and tumor suppressors, expressed in most senescent cells (31). Decreased expression of SIRT1 (a NAD-dependent deacetylase) has been associated with cellular senescence (50). Consistent with a senescent-associated phenotype, using a semiquantitative scoring method, we observed increased p16 and decreased SIRT1 expression in small airway epithelial cells from patients with COPD (Fig. 1*D*). No significant differences in p21 expression were observed (data not shown). Using immuno-FISH (p16 and γH2A.X), we found that p16-positive cells have more TAF than p16-negative cells (Fig. 1*E*), suggesting that TAF may be involved in senescence induction.

**Fig. 3.**
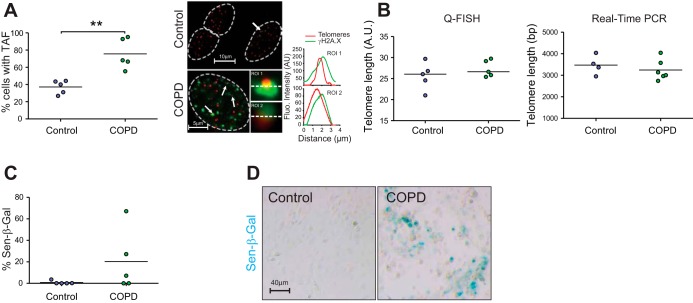
Cultured small airway epithelial cells from patients with COPD show increased TAF and senescence-associated β-galactosidase (Sen-β-Gal) activity without significant telomere shortening. Small airway epithelial cells (*passages 1–3*) from patients with COPD and aged-matched controls were stained for γH2A.X and telomeres by immuno-FISH. *A*, *left*: dot plots represent percentage of cells positive for TAF for each individual generated by quantifying *Z*-stacks of at least 40 cells per subject. Values are the mean for individual subjects, with the horizontal line representing group mean. *Right*: representative image of immuno-FISH staining for γH2A.X (green) and telomeres (red) in small airway epithelial cells from patients with COPD and controls captured using ×100 oil objective and following Huygens (SVI) deconvolution. Arrows point to γH2A.X foci colocalizing with telomeres (TAF), depicted by associated histograms and shown at higher magnification on the *right* (images are from one single *Z*-plane). *B*: dot plots representing telomere length quantified by Q-FISH (controls, *n* = 5; COPD, *n* = 5; *left*) and telomere length quantified by real-time PCR (controls, *n* = 4; COPD, *n* = 6; *right*). Horizontal line represents group mean. *C*: dot plot represents percentage of cells positive for Sen-β-Gal staining generated by quantifying at least 500 cells per subject. Dots represent mean value per subject, with horizontal line representing group mean. *D*: representative image of Sen-β-Gal staining (blue) in small airway epithelial cells from patients with COPD and controls. Statistics: independent samples *t*-test: **P < 0.05.

Following ex vivo analysis, we investigated whether TAF were increased in small airway epithelial cells isolated from the COPD lung (Table 2). By immuno-FISH, we found a significant increase in percentage of cells positive for TAF from patients with COPD (Fig. 3*A*), without significant differences in telomere FISH intensity (Fig. 3*B*). Because we found no significant differences in telomere length using Q-FISH, we compared telomere length in small airway epithelial cells isolated from COPD patients and age-matched controls (Table 3), using quantitative real-time PCR. Similarly, we detected no statistically significant differences (Fig. 3*B*). Small airway epithelial cells isolated from the COPD lung had increased positivity of Sen-β-Gal; however, this failed to reach statistical significance, with extensive interpatient variability observed (Fig. 3, *C* and *D*).

**Table 3.** Clinical characteristics of patients with COPD and controls (small airway epithelial cells used for RT-PCR)

	Patients with COPD	Normal Controls
*n*	6	4
Sex, male/female	5/1	3/1
Age, yr	54.6 ± 7	49.5 ± 7.59
FEV_1_, liter	0.54 ± 0.17‡	3.75 ± 1.24
FEV_1_, %	15.6 ± 14.13§	111 ± 17.33
FVC, liter	2.59 ± 0.95*	5.02 ± 1.96
FVC, %	58.8 ± 19.79†	120.25 ± 26.36
Smoking history, pack·yr	38.6 ± 21.67†	0
GOLD score, I/II/III/IV	0/0/0/0/6	0/0/0/0

Values are means ± SD; *n*, no. of subjects.

* *P* < 0.05,

† ^**^P < 0.01,

‡ *P* < 0.001,

§ *P* < 0.0001 compared with controls.

#### Telomere-associated foci increase in small airway epithelial cells in mice with age and following cigarette smoke exposure.

Following our observation that TAF were increased in small airway epithelial cells of patients with COPD, we investigated whether TAF increased in small airway epithelial cells during physiological aging. Mice have long telomeres and express ubiquitously the enzyme telomerase; hence it was believed that telomere dysfunction did not play a role in cellular senescence in murine tissues (39). However, our group demonstrated that TAF accumulate in liver and intestine with age (25), and TAF have been shown to quantitatively predict mean and maximum lifespan in both short- and long-lived mice cohorts (29).

**Fig. 4.**
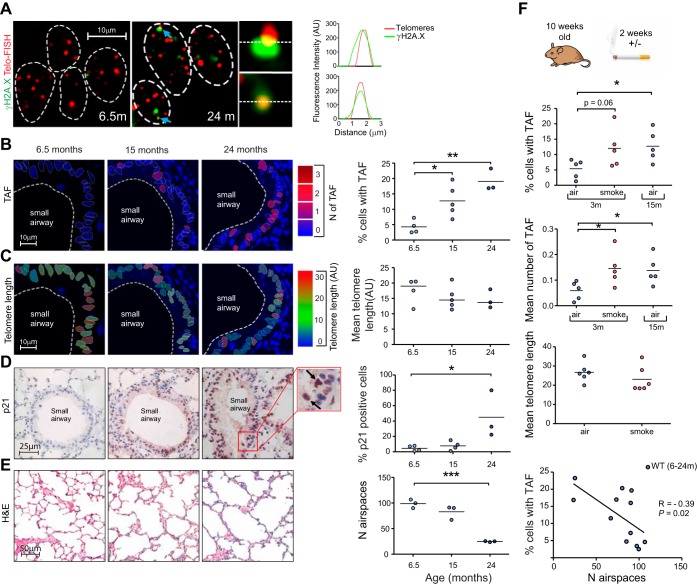
TAF increase in small airway epithelial cells in mice in vivo with age and following cigarette smoke exposure without significant telomere shortening. Lung tissue sections from male C57BL/6 mice of increasing age (6.5–24 mo) were stained by immunofluorescence and immunohistochemistry for senescence-associated markers. *A*: representative image of immuno-FISH for γH2A.X (green) and telomeres (red) in small airway epithelial cells from 6.5- and 24-mo-old mice captured using ×100 oil objective and following Huygens (SVI) deconvolution. Arrows point to γH2A.X foci colocalizing with telomeres (TAF), depicted by associated histograms and shown at higher magnification on the *right* (images are from one single *Z*-plane). *B*: immuno-FISH images of 6.5-, 15-, and 24-mo-old mice color-coded according to number of TAF (blue: low number; red: high number). *C*: Q-FISH images of 6.5-, 15-, and 24-mo-old mice color-coded according to telomere length (blue: short; red: long). Dot plots represent percentage of cells positive for TAF and mean telomere length for each animal generated by quantifying *Z*-stacks of at least 100 cells per animal. *D*: representative images of p21 immunostaining (brown) in small airway epithelial cells from 6.5-, 15-, and 24-mo-old mice captured using ×40 objective. Arrows point to p21-positive cells. Dot plots represent the percentage of epithelial cells expressing p21 in small airways generated by quantifying 10 random images for each animal. *E*: representative images of hematoxylin and eosin (H&E) staining of alveolar air spaces in 6.5-, 15-, and 24-mo-old mice captured using ×20 objective. Dot plots represent mean number of air spaces quantified from 10 random images taken for each animal. Correlation between mean number of TAF and number of air spaces in wild-type (WT) mice aged between 6.5 and 24 mo of age is shown. *F*: C57Bl/6 mice at 10 wk of age were exposed to cigarette smoke for 1 h, twice daily, or room air for 14 days, and immuno-FISH was carried out on sections from the left lung. Dot plots represent percentage of cells positive for TAF, mean number of TAF per cell, and mean telomere length of small airway epithelial cells for each animal, generated by quantifying *Z*-stacks of at least 100 cells per animal. Values are the mean for individual animals, with the horizontal line representing group mean. Statistics: independent samples *t*-test: *P < 0.05, **P < 0.01, ***P < 0.001.

We found a significant increase in percentage of cells positive for TAF from 6.5 until 24 mo of age (as well as mean number of TAF per cell, not shown) (Fig. 4, *A* and *B*). No significant changes in telomere FISH intensity were found; however, a tendency for decreased FISH intensity in older animals was observed (Fig. 4*C*).

Telomere dysfunction has been associated with increased expression of p21 (12). Consistently, we found with increasing age that a greater percentage of small airway epithelial cells stained positive for p21 (Fig. 4*D*). The aging lung is associated with structural changes similar to those that occur in emphysema, including distal air space enlargement (28). Consistent with this, we found increased air space size in mice with age, indicated by a decreasing number of air spaces per visual field (Fig. 4*E*). Interestingly, telomere FISH intensity did not correlate with air space number; however, there was an inverse correlation between percentage of cells positive for TAF and number of air spaces (*P* = 0.02) (Fig. 4*E*). These results suggest that telomere dysfunction may play a role in age-related lung tissue decline.

Cigarette smoke has been associated with early onset-senescence and induction of H2A.X phosphorylation in human pulmonary endothelial cells in vitro (3), and telomere length is reduced in small airway epithelial cells isolated from healthy smokers (49). However, the role of cigarette smoke in activation of a DDR specifically at telomeres has not been fully elucidated. We found that 3-mo-old mice exposed to cigarette smoke, twice daily for 2 wk, had an increased percentage of small airway epithelial cells positive for TAF, similar to levels observed in mice at 15 mo of age. While this increase was not significant (*P* = 0.06), we found that mean number of TAF increased significantly (*P* = 0.03) (Fig. 4*F*). No significant differences in telomere FISH intensity were observed (Fig. 4*F*). Altogether, these results suggest that small airway epithelial cells accumulate TAF with age, which can be accelerated by cigarette smoke exposure.

#### Late-generation TERC^−/−^ mice show increased telomere-associated foci (TAF) in small airway epithelial cells and early-onset emphysema.

**Fig. 5.**
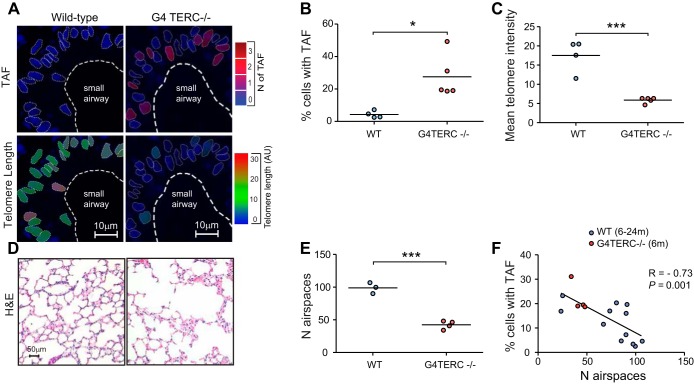
Late-generation *TERC*^*−/−*^ mice show increased TAF in small airway epithelial cells and early-onset emphysema. Immuno-FISH staining for γH2A.X and telomeres was carried out on lung tissue sections from late-generation *TERC*^*−/−*^ and WT C57Bl/6 mice at 6.5 mo old. *A*: immuno-FISH images of small airway epithelial cells from WT and late-generation [fourth generation (G4)] *TERC*^*−/−*^ mice color-coded according to number of TAF (blue: low number; red: high number) and telomere length (blue: short; red: long). Dot plots represent the percentage of cells positive for TAF (*B*) and the mean telomere length (*C*) for each animal generated by quantifying *Z*-stacks of at least 100 cells per animal taken using ×100 oil objective. *D*: representative images of H&E staining of alveolar air spaces in WT and G4 *TERC*^*−/−*^ mice captured using ×20 objective. *E*: dot plots represent the mean number of air spaces quantified per visual field from 10 random images taken for each animal. Values are the mean for individual animals, with the horizontal line representing group mean. *F*: correlation between percentage of small airway epithelial cells positive for TAF and number of air spaces. Statistics: independent samples *t*-test, Pearson's correlation: *P < 0.05, ***P < 0.001.

At late generations, mice deficient in the RNA component of telomerase (*mTERC*^−/−^) exhibit a number of phenotypes indicative of premature aging, thought to be due to early onset of senescence (8). Late-generation *mTERC*^−/−^ mice show critically short telomeres in most tissues and premature incidence of TAF. We found at 6 mo of age that G4 *mTERC*^−/−^ mice have an increase in percentage of small airway epithelial cells containing TAF and decreased telomere FISH intensity (Fig. 5, *A*–*C*). Consistent with the hypothesis that telomere dysfunction contributes to loss of alveolar integrity, we found a significant reduction in number of air spaces in G4 *mTERC*^−/−^ mice (Fig. 5, *D* and *E*). The correlation between TAF and number of air spaces we report is strengthened when G4 *mTERC*^−/−^ are added (Fig. 5*F*); however, telomere FISH intensity still does not correlate (not shown).

#### Cigarette smoke extract induces TAF and senescence markers in primary human airway epithelial cells and MRC5 fibroblasts.

In vitro exposure to cigarette smoke has been shown by several groups to result in expression of senescence-associated markers (3, 38). Nevertheless, the role of telomere dysfunction in cigarette smoke-induced senescence is less clear. Recent data from our group and others have revealed that stress-induced activation of a DDR at telomeres is persistent compared with nontelomeric damage, mostly because of inhibition of DNA repair mechanisms at telomere regions (20, 25). This suggests that TAF, given their persistence, may be excellent markers for age-related accumulated damage.

**Fig. 6.**
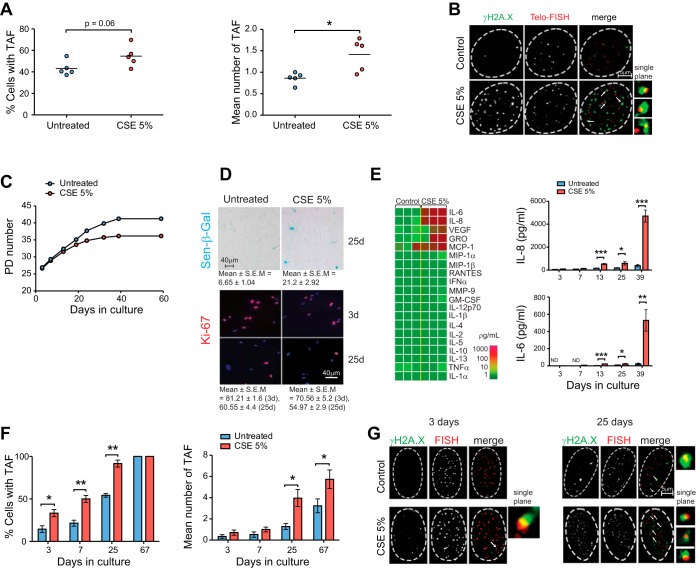
Cigarette smoke induces TAF and senescence markers in human MRC5 fibroblasts and primary small airway epithelial cells in vitro. *A*: normal human small airway epithelial cells (*n* = 5) were exposed to 5% cigarette smoke extract (CSE) (two exposures, 48 h apart) or left untreated and analyzed for γH2A.X and telomeres by immuno-FISH. Dot plots represent percentage of TAF-positive cells (*left*) and mean number of TAF (*right*) per cell generated by quantifying *Z*-stacks of at least 50 cells per subject using ×100 oil objective. Horizontal line represents group mean. *B*: representative *Z*-projection of immuno-FISH staining for γH2A.X (green) and telomeres (red) in small airway epithelial cells exposed to 5% CSE or untreated captured using ×100 oil objective and following deconvolution. Arrows point to γH2A.X foci colocalizing with telomeres (TAF) and are shown at higher magnification on the *right* (images are from single *Z*-plane). *C*: MRC5 cells (untreated or exposed to 5% CSE every 48 h) were subjected to repeated passaging and population doubling (PD) level calculated for each condition and plotted against number of days in culture. *D*: representative images of Sen-β-Gal staining in MRC5 cells after 25 days in culture, and representative images of Ki-67 staining in MRC5 cells after 3 and 25 days in culture. *E*, *left*: secreted protein array (RayBiotech) for range of inflammatory proteins 39 days following 5% CSE. Data are from *n* = 3 experiments. GRO, growth-related oncogene; MCP-1, monocyte chemotactic protein-1; MIP, macrophage inflammatory protein; RANTES, regulated on activation, normal T cell expressed and secreted; MMP-9, matrix metalloproteinase; GM-CSF, granulocyte-macrophage colony-stimulating factor. *Right*: concentrations of IL-6 and IL-8 in cell culture media from MRC5 cells exposed to 5% CSE or untreated were measured by ELISA and are presented as means ± SE of *n* = 4 experiments. *F*: immuno-FISH staining for γH2A.X and telomeres was carried out on MRC5 cells from a number of time points. Percentage of cells positive for TAF (*left*) or mean number of TAF per cell (*right*) generated by quantifying *Z*-stacks of at least 30 cells per condition using ×100 oil objective are shown. Values are means ± SE. *G*: representative *Z*-stacks of immuno-FISH staining for γH2A.X (green) and telomeres (red) in MRC5 cells exposed to 5% CSE or untreated at 3 and 25 days in culture captured using ×100 oil objective and following Huygens (SVI) deconvolution. Arrows point to γH2A.X foci colocalizing with telomeres (TAF), shown at higher magnification on the *right* (images are from single *Z*-plane). Statistics: independent samples *t*-test: *P < 0.05, **P < 0.01, ***P < 0.001.

To determine whether CSE induced TAF in isolated small airway epithelial cells, we cultured cells isolated from healthy nonsmoking controls (*n* = 5) and exposed them to 5% CSE for 48 h. Small airway epithelial cells had increased TAF following CSE exposure (Fig. 6, *A* and *B*); however, analysis of Sen-β-Gal expression revealed no significant increases (not shown). Our data shows that TAF are induced as a consequence of CSE and may precede the induction of other senescence markers.

Epithelial cells cannot be cultured for prolonged periods of time without induction of epithelial-to-mesenchymal transition, a process whereby epithelial cells lose their epithelial features and acquire mesenchymal characteristics. This limits our ability to determine the effects of chronic cigarette smoke exposure on telomere dysfunction and other senescence-associated phenotypes. Therefore, we used normal human fetal lung fibroblasts (MRC5), which can be cultured for longer periods of time and are not overly sensitive to the effects of cigarette smoke exposure. MRC5 cells were cultured for 60 days in the presence or absence of 5% CSE to determine the effects of long-term cigarette smoke exposure. Consistent with previous observations, we show that long-term exposure to CSE induces accelerated senescence in MRC5 cells, evidenced by reduced population doublings (Fig. 6*C*), increased Sen-β-Gal activity (Fig. 6*D*), and reduction in proliferation marker Ki67 (Fig. 6*D*). Senescence is characterized by increased secretion of bioactive, proinflammatory peptides; the so-called senescence-associated secretory phenotype (SASP). We first conducted a cytokine array analysis of 20 proinflammatory mediators and found that CSE exposure for 39 days leads to increased secretion of IL-6, IL-8, growth-related oncogene, monocyte chemotactic protein-1, and vascular endothelial growth factor (Fig. 6*E*). Most other cytokines or growth factors analyzed were secreted below detection level. This is consistent with previous reports suggesting that proinflammatory cytokines IL-6 and IL-8 are major components of the SASP and have been shown to contribute to the induction and maintenance of senescence in autocrine and paracrine fashions (1, 2). ELISAs for IL-6 and IL-8 detection independently confirmed the findings of the cytokine array (Fig. 6*E*). This rise in cytokine secretion following CSE exposure was first observed at *day 13*, but became enriched after 39 days in culture when cells reached premature senescence.

Consistent with a potential role for telomere dysfunction in the process, immuno-FISH revealed a significant increase in the percentage of cells containing TAF and in the mean number of TAF following CSE exposure, preceding other markers of senescence (Fig. 6, *F* and *G*). At *day 67* in culture, all cells were positive for TAF, but mean number of TAF was increased in CSE-exposed cells (Fig. 6*F*).

#### Cigarette smoke-induced proinflammatory phenotype is accelerated by ROS-dependent telomere dysfunction.

**Fig. 7.**
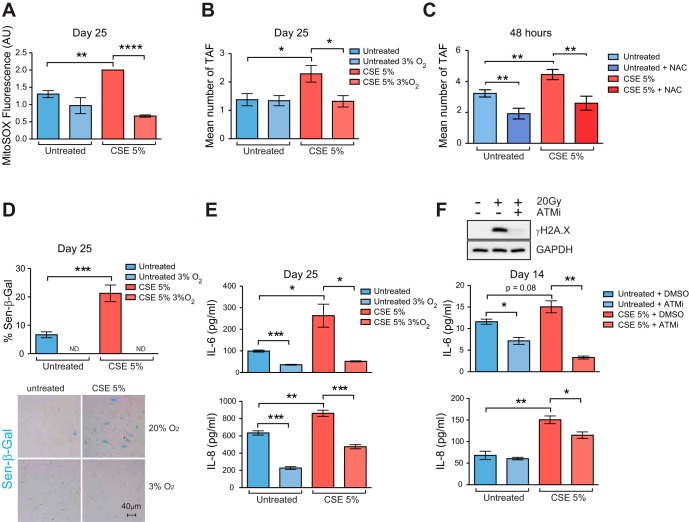
Cigarette smoke-induced proinflammatory phenotype is accelerated by ROS-dependent telomere dysfunction. MRC5 fibroblasts (PD20-25) were grown in culture for 25 days with 5% CSE, replaced every 48 h, or normal DMEM (untreated control) under normoxia (20% O_2_) or hypoxia (3% O_2_). *A*: MitoSOX staining coupled with FACS analysis was used to measure mitochondrial ROS generation after 25 days in culture. Bar graphs represent mean fluorescence intensity ± SE of *n* = 3 experiments. *B*: immuno-FISH staining for γH2A.X and telomeres was carried out after 25 days in culture. Bar graphs represent mean number of TAF per cell generated by quantifying *Z*-stacks of at least 50 cells per condition taken using ×100 oil objective, represented as means ± SE. *C*: primary human small airway epithelial cells were treated with 5% CSE for 48 h in the presence or absence of 2.5 mM *N*-acetyl-cysteine (NAC) compared with untreated cells. Values are means ± SE of 50 cells. *D*: bar graph and representative image of Sen-β-Gal staining (blue) after 25 days in culture. Bar graphs represent percentage of cells positive for Sen-β-Gal generated by quantifying at least 500 cells per condition. Values are means ± SE of at least 10 random planes. *E*: concentrations of IL-6 and IL-8 in cell culture media from MRC5 cells after 25 days in culture were measured by ELISA and are presented as means ± SE of *n* = 3 experiments. *F*: human MRC5 fibroblasts (PD20-25) were grown in culture for 14 days with 5% CSE, replaced every 48 h, or normal DMEM (untreated control) with DMSO or 10 μM KU55933 [ataxia telangiectasia mutated inhibitor (ATMi)]. Concentrations of IL-6 and IL-8 in cell culture media after 14 days in culture were measured by ELISA and are presented as means ± SE of *n* = 3 experiments. Representative example of Western blot shows reduced γH2A.X expression 1 day after 20 Gy X-ray irradiation of MRC5 cells when treated with the same concentration of the ATM inhibitor. Statistics: independent samples *t*-test: *P < 0.05, **P < 0.01, ***P < 0.001, ****P < 0.0001.

Mechanistically, it is unclear how CSE induces telomere dysfunction. Telomeres are highly sensitive to oxidative stress compared with the bulk of the genome and less efficiently repaired when subjected to single-strand breaks and DSBs (25, 42). Moreover, it has been shown that exposure to CSE increases ROS and oxidative stress markers in human fibroblasts and airway epithelial cells (9, 16). To determine whether TAF were induced by oxidative stress, we cultured MRC5 fibroblasts under low oxygen pressure (3% O_2_) and exposed them for 25 days to 5% CSE. Culturing cells at low oxygen pressure is an accepted method of reducing intracellular ROS, and under these conditions we and others have previously reported reduced mitochondrial and cellular oxidative stress (39, 40, 43), which resulted in reduced DNA damage and delayed onset of senescence. We found that CSE significantly increased mitochondrial-derived ROS (measured by MitoSOX fluorescence), which was suppressed when culturing cells under 3% O_2_ (Fig. 7*A*). Consistent with a causal role for oxidative stress in TAF induction, we found that low oxygen was able to suppress smoke-induced increases in TAF (Fig. 7*B*). Similarly, treatment of primary small airway epithelial cells with antioxidant *N*-acetyl-cysteine significantly reduced short-term CSE-induced increases in TAF (Fig. 7*C*). As shown previously, CSE exposure increased percentage of cells positive for Sen-β-Gal, however, cultivation of cells at 3% O_2_ drastically reduced frequencies of Sen-β-Gal-positive cells (Fig. 7*D*). Furthermore, we found that CSE-driven growth arrest was suppressed upon cultivation of MRC5 fibroblasts at low oxygen (not shown).

Telomere-dysfunction and resulting DDR activation result in increased expression of IL-6 and IL-8 (44). Consistent with a role for ROS-dependent telomere dysfunction contributing to the SASP, we found that low oxygen significantly reduced IL-6 and IL-8 secretion in MRC5 fibroblasts, irrespective of smoke exposure (Fig. 7*E*).

Mechanistically, it has been shown that persistent ATM activation is necessary for induction of the SASP (44). To test the hypothesis that CSE-dependent activation of a DDR results in increased IL-6 and IL-8, we treated smoke-exposed MRC5 fibroblasts with an ATM inhibitor (KU55933). We first demonstrated that KU55933 suppresses phosphorylation of γH2A.X (a target of ATM kinase), confirming its role in DDR inhibition (Fig. 7*F*). At 14 days, we found that ATM inhibition represses smoke-induced TAF increase (not shown), as well as IL-6 and IL-8 secretion (Fig. 7*F*), supporting the hypothesis that smoked-induced DDR activation results in increased SASP.

Altogether, the results from MRC5 cells suggest that cigarette smoke exposure causes telomere dysfunction, possibly through increased oxidative stress, which leads to senescence induction and SASP activation.

## DISCUSSION

Increased cellular senescence is a major feature of aging and has been implicated in COPD pathogenesis. Short telomeres, known activators of cellular senescence, have been implicated in COPD, mostly in circulating leukocytes (45).

Recent data has questioned the utility of telomere length in circulating leukocytes as a biomarker of aging. While some studies suggest that leukocyte telomere length may act as a proxy for telomere length in other somatic cell types, there is evidence suggesting that this is not true for some tissues (47). Furthermore, recent studies have suggested telomere dysfunction can be induced independently of length. In fact, data suggest that senescence can be induced by activation of a DDR at relatively long telomeres in human fibroblasts during stress-induced (20, 25), replicative (30), and oncogene-induced senescence in vitro (46) and in mice in vivo (25). While the mechanisms driving telomere dysfunction are still unclear, these data suggest that a “critical” telomere length may not be the sole determinant in the activation of a persistent DDR.

Using Q-FISH and real-time PCR in small airway epithelial cells, we failed to detect robust differences in telomere length between controls and patients with COPD. This contrasts with previous reports where telomere shortening is described in other lung cells from patients with COPD, including alveolar type II cells and endothelial cells (5, 48). It is possible that our study failed to detect differences in telomere length due to a relatively small sample size. Decreased telomere length in smokers (49) and patients with COPD (5) has been described, using larger sample sizes than in our study. However, only small differences in telomere length have been reported (<15% in most cases). We observe significant increases in the frequency of cells positive for TAF in patients with COPD, even with smaller sample sizes. Moreover, we have found clear evidence for increased TAF in small airway epithelial cells in lung tissue from COPD patients, which are younger than controls, demonstrating that TAF are robust indicators of COPD-associated damage, despite the age discrepancy. Consistently, another study also failed to find differences in telomere length between lung fibroblasts isolated from patients with emphysema and aged-matched controls, despite increased expression of senescence-associated markers (37). In addition to increased TAF, we observed increased p16, which is considered a hallmark of senescence. Moreover, TAF content was greater in p16-positive cells, suggesting that TAF correlate with expression of senescence markers. We did not detect differences in p21 positivity between patients with COPD and controls. However, the p16-pRB pathway may be activated following activation of, or independent to, the p21 pathway (24).

Our study cannot eliminate telomere shortening as a mechanism driving COPD-associated telomere dysfunction because *1*) in age-matched isolated small airway epithelial cells, our sample number is relatively small; and *2*) in lung tissue sections, where our numbers are greater, the controls were older. Nonetheless, comparison of individual telomere lengths colocalizing and not with γH2A.X in small airway epithelial cells present in COPD lung tissue revealed no significant differences, suggesting that telomeres can signal a DDR, irrespective of length. These data are in accordance with several recent publications reporting telomere dysfunction, irrespective of length, in a variety of cells (20, 25). We have used γH2A.X immunoreactivity alone to determine damage both in genomic DNA and at telomere regions. It has previously been shown that γH2A.X immunoreactivity can be detected independent of DNA damage. For example, one study described two distinct γH2A.X populations during cell division: one that formed large foci that colocalize with DSB repair proteins, and another forming abundant small foci dissociated from repair proteins, which may have a role in the mitotic process (35). It is, therefore, possible that the presence of γH2A.X foci at telomeres that we observe occur independently of oxidative DNA damage and DSBs to the sequence. However, based on our data, we have reasons to believe that the TAF we observe are not those described small foci associated with mitosis. First, γH2A.X foci, which generally colocalize with telomeres, are the largest in size, both in small airway epithelial cells and in human fibroblasts (25). Second, the presence of TAF inversely correlates with decreased proliferation and downstream pathways of senescence in both fibroblasts and small airway epithelial cells. However, since we have not analyzed colocalization between γH2A.X, telomeres, and DSB repair proteins, it is possible that the TAF we observe may not be the outcome of DSBs, but due to activation of another signaling event.

Our study suggests that cigarette smoke enhances oxidative stress and contributes to telomere dysfunction in vitro and in vivo. Data indicate that telomeres are particularly susceptible to oxidative stress compared with the rest of the genome (25, 42); however, the mechanisms are not completely understood. Telomere repeats contain guanine triplets, which are susceptible to oxidative modifications and are less efficiently repaired when subjected to different types of DNA damaging agents (20, 25). While cigarette smoke has been shown to induce γH2A.X (3), this is, to our knowledge, the first time TAF have been observed. The importance of this finding lies in the fact that, when a DDR is induced at telomeric regions, it is persistent and unresolved, which is characteristic of senescence (25). We did not determine whether cigarette smoke exposure increased levels of oxidative stress in vivo. However, it has been shown by other groups that both short- and long-term exposures to cigarette smoke increase markers of oxidative stress in the lungs of mice, including 8-hydroxy-2′-deoxyguanosine and 4-hydroxynonenal (6, 51, 52). The importance of oxidative DNA damage to the pathogenesis of COPD has been underscored by a number of studies showing that patients with COPD have different types of oxidative DNA damage in both the nuclear and mitochondrial genomes (7, 11, 33, 41). However, this is the first report, to our knowledge, describing possible oxidative damage to telomere regions (independently of telomere shortening) in the context of physiological lung aging and cigarette smoke-induced accelerated lung aging. While we do not disregard the role of other forms of oxidative damage in the pathogenesis of COPD or following cigarette smoke exposure, we hypothesize that telomere-associated damage is highly important in the context of senescence, since telomeres are particularly susceptible to oxidative damage and are irreparable.

Telomere length in COPD patients has been shown to inversely correlate with mRNA expression of inflammatory cytokines (5); however, it is still unclear whether there is a causal link between telomere dysfunction and the SASP as a result of cigarette smoke exposure. We demonstrate that *1*) inhibition of ROS suppresses smoke-induced telomere dysfunction, along with decreased secretion of IL-6 and IL-8; and *2*) inhibition of ATM, one of the main initiating factors of a DDR, diminishes CSE-induced IL-6 and IL-8 release. Altogether, these data suggest a causal link between ROS, activation of a DDR at telomeres, and the proinflammatory phenotype characteristic of senescence. However, it is not possible to delineate from these experiments whether telomeric damage specifically is responsible for ATM-dependent SASP induction, since ATM inhibition with KU55933 will have global effects. Technically, it would be very difficult to inhibit ATM activity only at telomere regions, but this would allow any causal link between telomere dysfunction and SASP activation to be identified. Moreover, it is not possible to extrapolate the findings from MRC5 cells to primary airway epithelial cells, as we were unable to culture these cells for longer than 5 days without development of epithelial-to-mesenchymal transition-related phenotypic changes, which is a limitation of our study.

Our data propose that TAF correlate with development of lung emphysema more strongly than telomere length in aging mice and could play a causal role in age-related lung degeneration, given that late-generation *mTERC*^−/−^ mice show early onset of emphysematous-like changes. There is still uncertainty regarding the role of telomere length in emphysema: a previous study using G4 *mTERC*^−/−^ mice failed to observe lung emphysema (4). However, the authors reported very small differences in telomere length of <15% between wild-type and G4 *mTERC*^−/−^ mice, in contrast to an almost fourfold difference we observed. This may explain the discrepancies in the data, as another study using G4 *mTERC*^−/−^ reported loss of alveolar integrity coupled with similar telomere signal loss, as observed in our mice (32).

In summary, while our data do not invalidate the role of telomere shortening in COPD-associated senescence, it suggests that TAF are a more robust marker of senescence in COPD, compared with telomere length. We observe increases in percentage of cells positive for TAF in COPD, despite relatively small samples sizes. Moreover, although we observed good associations between number of γH2A.X foci alone and mean number of TAF with age and in the context of cigarette smoke exposure, we consistently observe more significant increases in TAF. Telomeres occupy just 0.02% of the genome, and thus the probability of damage occurring at telomeres is extremely low. Despite this, we observed robust increases in TAF with age and even following short-term cigarette smoke exposure, suggesting that telomeres may have particular properties that render them susceptible to damage. In fact, it has been shown that telomeres accumulate more single-stranded breaks than the bulk of the genome in response to oxidative stress (42). It has been argued that this may be due to the fact that telomeric repeats contain guanine triplets, which are remarkably sensitive to oxidative modifications (22). These factors, coupled with the reported protection of telomeres from repair activities, may contribute to their specific targeting and persistent damage as a consequence of cigarette smoke exposure and during the aging process. Further work needs to be performed to establish whether TAF are associated with COPD susceptibility and severity or have any prognostic value. We propose that TAF may be causal to structural decline and increased inflammatory processes that occur during physiological lung aging and in COPD.

## GRANTS

The research was funded/supported by the National Institute for Health Research (NIHR) Biomedical Research Centre for Ageing and Chronic Disease based at Newcastle Upon Tyne Hospitals National Health Service (NHS) Foundation Trust. J. Birch is funded partly by a UK Medical Research Council (MRC) studentship. N J. Green and E. Moisey are funded by the Welcome Trust. A. J. Fisher is partly supported by the COPD MRC/Association of the British Pharmaceutical Industry (COPDMAP) consortium. Work in J. F. Passos' laboratory is supported by a Biotechnology and Biological Sciences Research Council (BBSRC) David Phillips Fellowship and a BBSRC grant BB/K017314/1. A. De Soyza acknowledges a Higher Education Funding Council for England senior lectureship and the support of Northumbria Tyne and Wear Comprehensive local research network (NIHR-Clinical Research Network).

## DISCLAIMERS

The views expressed are those of the author(s) and not necessarily those of the NHS, the NIHR, or the Department of Health.

## DISCLOSURES

No conflicts of interest, financial or otherwise are declared by the author(s).

## AUTHOR CONTRIBUTIONS

Author contributions: J.B., C.C.-M., A.J.F., A.D.S., and J.F.P. conception and design of research; J.B., R.K.A., C.C.-M., G.H., F.M.M., N.J.G., and E.M. performed experiments; J.B., R.K.A., C.C.-M., G.H., F.M.M., and J.F.P. analyzed data; J.B., R.K.A., G.H., A.J.F., A.D.S., and J.F.P. interpreted results of experiments; J.B., R.K.A., D.J., and J.F.P. prepared figures; J.B. and J.F.P. drafted manuscript; J.B., R.K.A., C.C.-M., D.J., G.H., F.M.M., N.J.G., E.M., M.A.B., M.G.B., F.B., J.J.T., A.J.F., A.D.S., and J.F.P. edited and revised manuscript; J.B., D.J., A.J.F., A.D.S., and J.F.P. approved final version of manuscript.
